# The Promise of Liquid Biopsy to Predict Response to Immunotherapy in Metastatic Melanoma

**DOI:** 10.3389/fonc.2021.645069

**Published:** 2021-03-18

**Authors:** Luigi Fattore, Ciro Francesco Ruggiero, Domenico Liguoro, Vittorio Castaldo, Angiolina Catizone, Gennaro Ciliberto, Rita Mancini

**Affiliations:** ^1^ SAFU Laboratory, Department of Research, Advanced Diagnostics and Technological Innovation, Translational Research Area, IRCCS Regina Elena National Cancer Institute, Rome, Italy; ^2^ Department of Experimental and Clinical Medicine, University “Magna Graecia” of Catanzaro, Catanzaro, Italy; ^3^ Department of Clinical and Molecular Medicine, Sapienza University of Rome, Rome, Italy; ^4^ Department of Anatomy, Histology, Forensic- Medicine and Orthopedics, Sapienza University of Rome, Rome, Italy; ^5^ Scientific Directorate, IRCSS Regina Elena National Cancer Institute, Rome, Italy

**Keywords:** melanoma, immunotherapy, drug resistance, liquid biopsy, biomarkers

## Abstract

Metastatic melanoma is the deadliest form of skin cancer whose incidence has been rising dramatically over the last few decades. Nowadays, the most successful approach in treating advanced melanoma is immunotherapy which encompasses the use of immune checkpoint blockers able to unleash the immune system’s activity against tumor cells. Immunotherapy has dramatically changed clinical practice by contributing to increasing long term overall survival. Despite these striking therapeutic effects, the clinical benefits are strongly mitigated by innate or acquired resistance. In this context, it is of utmost importance to develop methods capable of predicting patient response to immunotherapy. To this purpose, one major step forward may be provided by measuring non-invasive biomarkers in human fluids, namely Liquid Biopsies (LBs). Several LB approaches have been developed over the last few years thanks to technological breakthroughs that have allowed to evaluate circulating components also when they are present in low abundance. The elements of this so-called “circulome” mostly encompass: tumor DNA, tumor and immune cells, soluble factors and non-coding RNAs. Here, we review the current knowledge of these molecules as predictors of response to immunotherapy in metastatic melanoma and predict that LB will soon enter into routine practice in order to guide clinical decisions for cancer immunotherapy.

## Background

**Figure 1:**
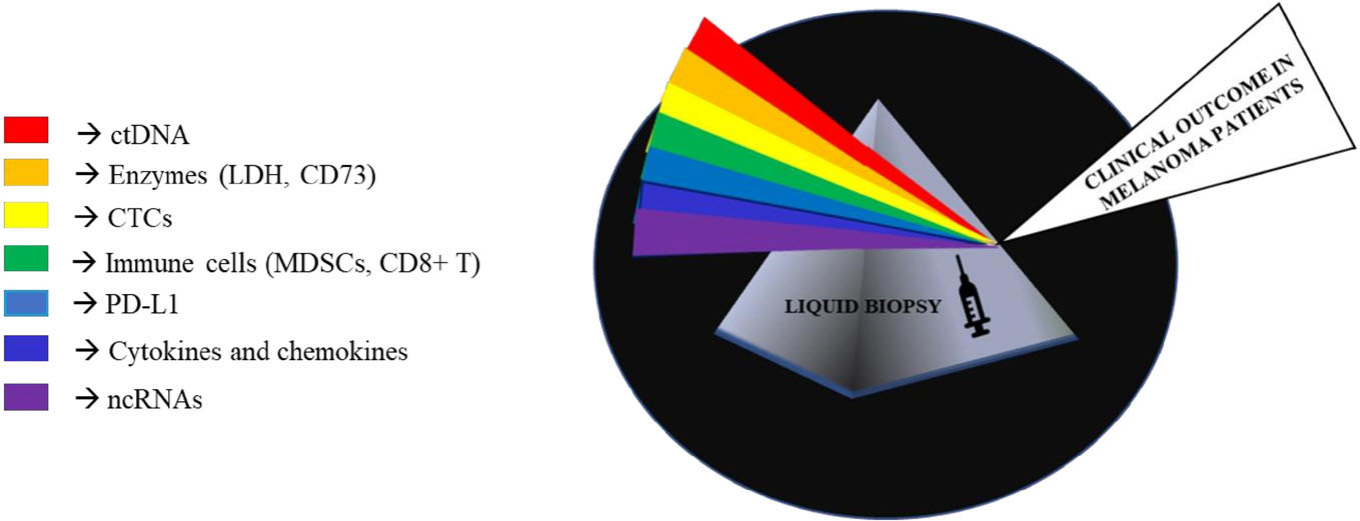
Representative image of non-invasive biomarkers available in liquid biopsies to manage metastatic melanoma.

Melanoma is a highly malignant tumor originating from melanocytes and is characterized by high metastatic propensity and high mortality rates (1). The development of melanoma involves several dynamic processes in which the immune system plays a key role (2). It is well known that tumor cells activate different mechanisms to escape immune surveillance: a process known as “cancer-immunoevasion” (1). In this review, we will not probe into describing these complex processes that are the focus of several other excellent reviews (1, 3–6). The most effective therapeutic approach for advanced melanoma relies on the possibility to reactivate immune cells on recognizing tumor cells as foreign components and on controlling their growth. This concept lies at the basis of immuno-oncology. Historically, this approach has been pursued through the use of interferon and interleukin cytokines (like IL-2), but clinical benefits were very modest and laden by substantial toxicities (7). However, the experiences derived from those drugs paved the way for more successful immunotherapies. The first of them was the development of ipilimumab as an anti-CTLA-4 antibody which was then later on followed by the discovery of inhibitors of PD-1/PD-L1 receptor-ligand couple (3, 7). These monoclonal antibodies, initially nivolumab and pembrolizumab and subsequently atezolizumab and durvalumab, represent the main therapeutic breakthroughs over the last few years (8). These molecules have radically changed the therapeutic scenario contributing to increasing overall survival not only in patients with metastatic melanoma but also in a growing number of cancer types (3, 7, 9). Furthermore, due to their non-overlapping mechanisms of action, combinations of nivolumab together with ipilimumab led to longer progression-free survival and a higher proportion of objective response rate as compared to monotherapies (10, 11). Unfortunately, cancer immunotherapy with checkpoint inhibitors is efficacious only in a subset of cases because 40–60% of patients do not achieve any significant therapeutic benefit (12, 13). In this scenario, it would be important to develop biomarkers capable of predicting response to immunotherapy, also in light of the elevated costs as well as the high degree of toxicity and severe adverse events of this type of therapy. Furthermore, it is worth considering that the therapeutic landscape of metastatic melanoma has been also improved by the availability of target therapy based on the combination of BRAF and MEK inhibitors in approximately 50% of patients carrying mutations in the amino acid V600 of the BRAF oncogene (14). Hence, in this subset of patients the choice of first line therapy, i.e. target therapy with BRAFi/MEKi vs. immunotherapy is still highly debated. These lines of evidence taken together stress the need to identify novel biomarkers that are able to predict the response to a given treatment. In the last few years, the use of liquid biopsies has provided major opportunities(LBs) (15, 16). LBs are a non-invasive approach which agreeably complement tumor biopsies in acquiring important information about tumor progression and response to therapy. Among the main advantages of LBs there is the possibility to follow disease evolution over time by collecting longitudinal sampling during the course of disease as well as during therapy. LBs can be represented by several human fluids, among them the most commonly evaluated is the blood (17). They are a source of different biological circulating tumor elements (defined “circulome”) that include proteins, circulating tumor DNA and RNA (ctDNA and ctRNA), circulating tumor cells (CTCs) and extracellular vesicles (EVs) (18, 19). Interestingly, these elements could derive from cancer cells themselves as well as from the tumor microenvironment. During the last few years thanks to significant technological advances and improvements in sensitivity, LBs have become a valuable tool from both diagnostic and prognostic points of view not only in melanoma but also across other cancer types. In this narrative review, we will focus on the state-of-the-art of liquid biopsies as predictors of response to immunotherapy in metastatic melanoma. In particular, we will focus on three main aspects a) circulating tumors DNA, b) circulating tumor cells (CTCs)/immune cells and soluble factors and c) non coding RNAs. These analytes are represented in **Figure 1**.

## Circulating Tumor DNA (ctDNA)

ctDNA is one of the most reliable biomarkers available in LBs (20) and its potential to predict response to immune checkpoint blockers (ICB) in melanoma has been investigated in several relevant studies over the last few years. They will be summarized in this section. From a biological point of view, ctDNA is constituted by DNA fragments released into the bloodstream by apoptotic or necrotic cancer cells and its amount in general adequately correlates with tumor stage and prognosis (21–24). The absolute amount of ctDNA varies along with the number of cancer cells and the total tumor burden. Furthermore, its evaluation is able to provide information on the genetic mutational profile of the tumor and can be a mirrored image of the heterogeneous complexity of advanced metastatic cancer (20). Besides ctDNA, the presence of cell-free DNA (cfDNA), a broader term that describes DNA that circulates freely in the bloodstream, but is not necessarily of tumor origin must also be reported (25). However, the evaluation of cfDNA, unlike ctDNA has not yet been considered a prognostic parameter *per se.*

The dosage of ctDNA has been made possible in the last decade thanks to modern advances in genomic and non-genomic technologies. One of the main issues to overcome is the limitation to detect ctDNA concentrations that are in general very low in the blood. In recent years, very sensitive multi-gene testing panels have been developed to measure ctDNA in LBs. Recently the FoundationOne Liquid CDx (https://www.foundationmedicine.com/test/foundationone-liquid-cdx) and Guardant360 CDx (https://guardant360cdx.com/) have recently been approved by the FDA for comprehensive tumor mutation profiling across solid cancers through LB sampling (26). In addition to large NGS panels, one of the most reliable methods in detecting ctDNA is through the use of Droplet Digital PCR (ddPCR) (27). This technology is able to provide ultrasensitive and absolute nucleic acid quantification and is particularly useful for low-abundant targets and for the design of patient-specific customized tests. Nowadays, the most accepted metric system for measuring ctDNA in LBs is to assess tumor-specific variant as copies/ml plasma through ddPCR.

Another potential biomarker of response to immunotherapy in melanoma is the emerging tumor mutation burden (TMB). This arises from the assumption that melanoma carries one of the highest mutational loads among human tumors. Even though TMB has been proposed as an independent predictor of response to immunotherapy (28), the most promising results have been obtained in combination with plasmatic ctDNA evaluation. This was the focus of the following study. In particular, it has taken advantage of a tumor panel comprised of 710 tumor-associated genes to reliably calculate TMB in liquid biopsies deriving from 35 melanoma patients treated with ipilimumab (as anti-CTLA-4) and nivolumab (as anti-PD-1) (27). The patients enrolled in this prospective study were almost equally divided into BRAF mutated (n=16) and BRAF wild type (n=19). The results demonstrated that TMB in the tumor biopsy was significantly higher (TMB > 23.1 Mut/Mb) in responders than in non-responders (TMB ≤ 23.1 Mut/Mb) before starting therapy (27). Furthermore, the same authors also evaluated TMB in combination with ctDNA (measured as copies/ml plasma) in the same experimental cohort. Their results demonstrated that the simultaneous reduction of both these parameters after 3 weeks of starting treatment was able to better distinguish patients who respond to combined immunotherapy (27). In particular, these authors measured as ctDNA markers not only mutated driver genes such as BRAF and NRAS, but also additional somatic variants like CDK4, GNAQ, STAT1 and others. It is important to point out that one limitation of this study relies on the fact that only a part of the patients enrolled (i.e. 63%) had ipilimumab or nivolumab as their first line systemic treatment whereas 13 patients received targeted therapy or PD-1 antibodies before.

The importance of ctDNA in predicting response to immunotherapy has also been demonstrated in another prospective study carried out by Seremet and colleagues (29). This study tested plasma samples from 85 patients undergoing anti-PD-1 therapy evaluating BRAF^V600E/K^ or NRAS^Q61/G12/G13^ in ctDNA (copies/ml plasma). Patients with undetectable ctDNA at baseline showed a better overall and progression free survival (OS and PFS) as compared to those patients with detectable ctDNA (29). Along the same line, another prospective study demonstrated that the assessment of ctDNA at baseline and during therapy was predictive for tumor response and clinical outcome in metastatic melanoma patients with a BRAF^V600E/K^ or NRAS^Q61/G12/G13^ mutation. In particular, the levels of ctDNA were evaluated in a prospective cohort of 40 advanced melanoma patients subjected to PD-1 inhibitors alone or in combination with ipilimumab at baseline and early during therapy. The objective was to assess the potential of ctDNA in predicting response and clinical outcome. Results showed that patients with higher basal levels of ctDNA (copies/ml plasma) and a persistently elevated ctDNA levels during therapy had a worse progression free survival (PFS) and overall survival (OS) (30). Similar results were achieved also in another prospective study which evaluated the clinical validity of ctDNA before and during ICB treatment (i.e. pembrolizumab) as a prognostic and predictive tool (31). In this prospective phase II study, ctDNA was measured in five distinct cohorts of patients with advanced solid tumors such as high-grade serous ovarian cancer (HGSOC), malignant melanoma and mixed solid tumors (MST) (31). ctDNA levels were measured in 316 plasma samples before (at basal levels) and during treatment with pembrolizumab. In particular, an early reduction in ctDNA (measured as mean tumor molecules per mL of plasma; MTM/mL) after two cycles of pembrolizumab and on-treatment ctDNA clearance distinguished patients with good prognosis independently from tumor types.

Along the same line, another study (32) assessed the levels of ctDNA (BRAF^mut^ or NRAS^mut^) as an indicator of response during anti-PD-1 treatment. In particular, these authors observed that the reduction of ctDNA levels in the blood after 2-3 weeks upon the first administration of nivolumab predicted the best response. In contrast, the anti-PD-1 antibody was ineffective in those patients where ctDNA did not decrease after starting treatment (32). This study also highlighted how ctDNA is a better predictor of response to therapy compared to other well-known clinical parameters such as Lactate Dehydrogenase (LDH).

Altogether these studies clearly demonstrated that ctDNA is a novel parameter in assessing response to immunotherapy both at baseline and during treatment and pave the way to propose the clinical practice of the ctDNA-based surveillance in melanoma patients treated with immune-checkpoint inhibitors.

Finally, ctDNA is useful in addition to being a novel parameter for assessing response to targeted therapy (BRAF/MEK inhibitors). Indeed, several studies have demonstrated that the fluctuations of this biomarker were generally correlated with treatment response to such inhibitors (33, 34). For example, Schreuer et al. analyzed BRAF^V600^ ctDNA in liquid biopsies deriving from 36 melanoma patients before and during treatment with dabrafenib and trametinib. Most of these samples showed detectable levels of ctDNA at baseline (75%) which rapidly decreased upon initiating targeted therapy and became undetectable in about 50% of those patients after 6 weeks of treatment (34). Most importantly, 27 out of 36 patients underwent disease progression (PD) and this was associated with an increase of ctDNA levels.

In summary, the results of this section (also summarized in **Table 1**) if further validated in larger prospective Phase III studies, provide the rationale for monitoring ctDNA at basal level to better stratify melanoma patients capable of responding to immunotherapy or also during administration of checkpoint inhibitors in order to early identify non-responding patients and stop therapy.

**Table 1:** List of predictive biomarkers of response to immunotherapy in metastatic melanoma coming from liquid biopsies.

LIQUID BIOPSY SOURCE	REFERENCES	N° ENROLLED PATIENTS/TREATMENTS	MARKERS	MAIN RESULTS
ctDNA	Forschner et al. (27)	- 35 treated with ipilimumab and nivolumab: - 16 BRAF mutated - 19 BRAF wild type	- *BRAF V600-* *- NRAS Q61-* - *CDK4, GNAQ, STAT1*	ctDNA levels is associated with tumor mutation burden (TMB)
ctDNA	Seremet et al. (29)	- 85 treated with pembrolizumab - 63 BRAF V600 mut - 22 NRAS mut	*- BRAF* V600E/K- *- NRAS* Q61/G12/G13	Undetectable pre-treatment ctDNA levels correspond to better OS and PFS
ctDNA	J H Lee et al. (30)	- 86 total: - 50 received anti PD-1 (pembrolizumab or nivolumab) - 36 received anti-PD1 + ipilimumab	*- BRAF* V600E/K- *- NRAS* Q61/G12/G13	Higher basal levels of ctDNA match up with poor prognosis
ctDNA	Bratman et al. (31)	- Pembrolizumab-treated Melanoma cohort (n=12)	- Personalized ctDNA based on 16 clonal somatic mutations	Baseline ctDNA levels correlate with PFS, OS, clinical response and clinical benefit
ctDNA	Ashida et al. (32)	5 treated with nivolumab: - 4 BRAF V600- mutated - 1 NRASQ61K	*BRAF* V600E/K NRAS Q61K	Reduction of ctDNA levels during treatment is predictive of best response to therapy
Circulating Tumor Cells	Hong et al. (35)	- 49 total - 33 treated with pembrolizumab - 16 treated with ipilimumab	Circulating tumor cells (CTCs) number	Low CTCs number during treatment improve PFS
Circulating Tumor Cells Soluble plasma protein	Lin et al. (36)	-52 total - 36 pembrolizumab - 5 ipilimumab - 8 nivolumab - 2 combination with ipilimumab/nivolumab 1 – ipilimumab/pembroluzumab	- CTCs number - Lactate dehydrogenase (LDH) levels	“Non-responders” patients are correlated to elevated CTCs number and LDH levels.
Circulating Tumor Cells	Khattak et al. (37)	- 40 treated with pembrolizumab	Identification of PDL-1 on CTCs	Melanoma patients with CTCs/PD-L1+ had better PFS in response to pembrolizumab
Soluble plasma proteins	Zhou et al. (38)	- 42 treated with ipilimumab plus bevacizumab - 23 ipilimumab - 35 pembrolizumab	Soluble PDL-1	Higher sPD-L1 levels at baseline had poorer in response to ipilimumab-based therapy.
Soluble plasma proteins	Weber et al. (39)	- 119 nivolumab treated melanoma patients - 101 nivolumab + pembrolizumab - 48 ipilimumab - 21 ipilimumab + nivolumab	209 circulating plasma protein. Most of which are involved in of acute phase of inflammation, complement activation and wound healing phenotypes.	High levels of identified 209 circulating plasma protein are correlated with worst response to immune-checkpoint inhibitors.
Soluble plasma proteins	Lim et al (40).	- 98total: - 40 anti-PD1 - 58 anti-CTLA-4 + anti-PD1	11 circulating cytokines (G-CSF, GM-CSF, Fractalkine, FGF-2, IFNα2, IL12p70, IL1a, IL1B, IL1RA, IL2, and IL13)	- Increased levels of 11 circulating cytokines are associated with the development of high-grade immune-related toxicity - TNF-a, IL-8 or IP-10 increase in non-responder patients to immunotherapy
Soluble plasma proteins	Sanmamed et al. (41)	- 29 treated with nivolumab and pembrolizumab - 19 treated with nivolumab and pembrolizumab	IL-8	Increased levels of IL-8 match up with worst response to immunotherapy
Soluble plasma proteins	Morello et al. (42)	- 37 treated with nivolumab and ipilimumab	Soluble CD73	Elevated levels at baseline of CD73 correlate with lower response rate, shorter survival and higher rates of progression disease (PD)
Lymphocytes	Capone et al. (43)	- 100 nivolumab-treated melanoma patients	CD8+ CD73+ subset lymphocytes	High presence of CD8+CD73+ lymphocytes correlate with worse response to immunotherapy
Myeloid-derived suppressor cell (MDSc)	Meyer et al. (44)	- 49 treated with ipilimumab	Myeloid-derived suppressor cell (MDSc) levels	Lower baseline levels of MDSC correlate with better response to ipilimumab
Myeloid-derived suppressor cell (MDSc)	Tarhini et al. (45)	- 35 treated with ipilimumab	Myeloid-derived suppressor cell (MDSc) levels	Low MDSCs levels predicts better PFS after neoadjuvant ipilimumab treatment
Neutrophil and lymphocyte	Capone et al. (46)	- 97 treated with nivolumab	Neutrophil to lymphocyte ratio (NLR)	Elevated NLR at baseline was associated with worst OS, PFS, and clinical response to immunotherapy
Extracellular Vesicles (EVs) Non coding RNAs	Huber et al. (47)	87 total; - 49 received nivolumab or ipilimumab	miR-146a, miR-155, miR-125b, miR-100, let-7e, miR-125a, miR-146b, miR-99b	High levels of specific microRNAs signature is related to not response to immunotherapies
Exosomes Non coding RNAs	Vignard et al. (48)	No patients were enrolled	miR-181, miR-498	miR-181-miR-498 impact on immune response through TNFa down-regulation
Extracellular Vesicles (EVs) Non coding RNAs	Shi et al. (49)	- 50 treated with immune checkpoint inhibitors	miR-551a, miR-4519 and miR-4674	-High levels of miR-551a were found in extracellular vesicles (EVs) of non-responder patients - High levels miR-4519 and miR-4674 were found in EVs of responder patients to immunotherapy
Non coding RNAs	Bustos et al. (50)	- 47 total -31 received Anti-PD1 -16 received Anti-PD1 plus Anti-CTLA4	miR-4649-3p, miR-615-3p and miR-1234-3p, miR-615-3p	- miR-4649-3p, miR-615-3p and miR-1234-3p signature are up-regulated in responder patient - miR-615-3p increased levels correlate with progression disease (PD)

OS, Overall Survival; PFS, Progression Free Survival; PD, Progression Disease; CTCs, Circulating Tumor Cells; LDH, Lactate Dehydrogenase; MDSc, Myeloid-derived suppressor cells; EVs, Extracellular Vesicles.

## Circulating Tumor Cells (CTCs), Soluble Factors and Immune Cells

LBs can be interrogated both for cellular and non-cellular components which can be exploited as promising biomarkers for monitoring and predicting response to immunotherapy in metastatic melanoma. This paragraph will focus on these aspects.

CTCs are cells that derive from primary/metastatic tumor sites, whose presence within the peripheral blood can predict the development of new metastatic lesions (51). During the last few decades, CTCs demonstrated to be suitable non-invasive biomarkers for studying the response to different types of therapies in melanoma in several studies, including target therapies and immunotherapies. One must consider that CTC reliability is challenged by the lack of standardized methodologies for their evaluation as well as for inter-patient heterogeneity that is responsible for the different levels of detectability of these cells in the blood (52). The methods for CTCs enrichment from peripheral blood are mainly divided into two categories: 1) enrichment for physical properties, i.e. size, and 2) enrichment for specific markers (53). The first method is based on microfilters that separate CTCs from other blood cells such as leukocytes. This is the approach recently used by our group. Briefly, we took advantage of ScreenCell^®^ size exclusion technology to isolate CTCs from blood samples taken from melanoma patients. In this way, cells entrapped on absorbent membrane can be processed by immunofluorescence for specific markers of interest. In our study, we demonstrated that increased phosphorylation of the ErbB3 receptor on CTC surface early occurs upon patient treatment with BRAFi and MEKi. Importantly, the activation of this receptor has been identified as an early mechanism of escape from therapies in melanoma. These findings open the possibility to further investigate the role of CTCs as predictors of response to targeted therapy (54).

The second approach to quantify CTCs was based on the detection of specific markers on their surface like MCSP, MCAM or MLANA (55). Taking advantage of this approach, CTCs were evaluated in the blood from a prospective cohort of 49 melanoma patients treated with immune checkpoint inhibitors (16 treated with ipilimumab, 33 with pembrolizumab). Interestingly, authors observed a strong decrease in CTC score within 7 weeks of therapy correlating with marked improvement in progression-free and overall survival (35).

Furthermore, the study of CTCs can also take advantage of the recent advent of genomic and non-genomic technologies in order to further increase sensitivity (55, 56). For example, in a recent study CTCs deriving from melanoma patients were identified using a multi-marker immunostaining panel for melanocytic proteins like gp100, S100 and MLANA. Thanks to this approach, CTCs were identified in the blood deriving from 52 melanoma patients who received immune checkpoints and their number positively correlated with high LDH levels. Thereby, authors designed a “disease outcome panel,” in which “high-risk” and “low-risk” melanoma patient subgroups were defined. Accordingly, patients belonging to the first subgroup were characterized by worse disease-free and overall survival (36).

These findings suggest that a valuable tool for evaluating response to immune-checkpoint inhibitors is the association between CTCs and LDH. LDH evaluation in the blood is one of the longstanding biomarkers for cancer progression and response to therapy in human cancers (57). Recently, the levels of this enzyme have also been tested for predicting response to immunotherapy in melanoma. For instance, in the recent Checkmate-067 study, patients treated with nivolumab were still alive at 4 years if the baseline LDH values were lower than those compared to patients who had higher levels before starting therapy, showing a worse overall survival (58).

Even though a significant proportion (about 27%) of PD-L1 negative melanoma patients may benefit from anti-PD-1/PD-L1 antibodies (59), its evaluation through immunohistochemistry (IHC) from tissue sections has been historically considered one of the most valuable biomarkers capable of predicting response to immunotherapy (60). However, this practice is limited to the primary tumors rendering the longitudinal sampling to monitor disease progression and response to therapy very difficult.

From here on, a potential biomarker of response to anti-PD-1/anti-PD-L1 therapy can be considered the expression of PD-L1 on CTC surface, as proposed for breast cancer, non-small cell lung cancer (NCLS) and also metastatic melanoma (61, 62). Initial findings promoted the activation of prospective clinical trials testing PD-L1 expression on CTCs for monitoring the response to immune-checkpoint inhibitors. For instance, it has been demonstrated that melanoma patients with high levels of CTCs that are positive for PD-L1 had better progression-free survival (PFS) after treatment with pembrolizumab as compared to patients with high CTCs with low PD-L1 levels. Furthermore, in the same study it was also shown that the ratio of PD-L1+/PD-L1- CTCs decreases upon treatment in responder patients whereas increases or remains unchanged in most non-responders. These data suggest that the detection of those cells may help stratify responder and non-responder patients to immunotherapy (37). The limitation of this pioneering study is related to the small cohort of patients tested and highlights the need to strengthen those findings in larger studies. However, because CTCs are usually very rare, fragile and difficult to capture, PD-L1 assessment on their surface remains a technical challenge.

A second method used to evaluate PD-L1 as a biomarker of response to immunotherapy relies on the discovery of its existence in a soluble form in the blood, known as sPD-L1 (63). This molecule has been identified in different human cancers, including renal cell carcinoma, multiple myeloma, large B-cell lymphoma and also in melanoma (64–66). In this context, Zhou and colleagues have demonstrated that melanoma patients with higher sPD-L1 levels at baseline had poorer outcome as compared to those patients with moderate/low sPD-L1 in response to ipilimumab-based therapy (38). In this context, *in vitro* studies have highlighted that sPD-L1 retains the signaling domain necessary for interacting with PD-1 on T cells and delivering immune-inhibitory signals (67). This could be the reason why, in the same study, a further increase of sPD-L1 levels in the blood has been observed in those patients who did not respond to immunotherapy as compared to those that did (responders).

Besides PD-L1, also different chemokines and cytokines have been proposed to be able to predict response to immunotherapy in melanoma in LBs. For example, due to the use of MALDI-TOF mass spectroscopy, a panel of 209 serum proteins that are associated with a better response to immune-checkpoint inhibitors, nivolumab and pembrolizumab has been identified (39). These circulating proteins, such as C reactive protein, serum amyloid A and P and angiostatin A are the indicators of a massive involvement of acute phase response, inflammation, complement activation and wound healing.

The detection of specific chemokines and cytokines in the blood has also been correlated to immune-mediated toxicity, an adverse event that requires interrupting immunotherapy treatment in melanoma patients. Along the same line, another study tested the expression levels of 65 cytokines in longitudinal plasma samples collected from melanoma patients treated with nivolumab or pembrolizumab alone or in combination with ipilimumab (40). Results showed that the increased levels of 11 circulating cytokines, such as IL1a, IL2, and IFNα2 were significantly associated with the development of high-grade immune-related toxicity. Furthermore, the same authors also demonstrated that other cytokines, like TNF-a, IL-8 or IP-10 increased their levels in the blood in patients who do not respond to immunotherapy as compared to those who did (responders).

The increased levels of IL-8 as a negative biomarker of response to either anti-PD-1 and anti-CTLA-4 was confirmed in another independent study carried out on metastatic melanoma (n=29) and NSCLC (n=19) patients (41).

Furthermore, recent studies have also focused on circulating levels of the CD73 enzyme which is expressed by different cellular populations of the tumor microenvironment such as the cancer cells themselves, endothelial and immune cells (68). This enzyme facilitates the establishment of a immunosuppressive tumor microenvironment inhibiting NK and T cell-mediated anti-tumor responses through producing extracellular adenosine (69). Increased levels of CD73 have been associated with worse prognosis in many types of cancers, including melanoma (70). In this context, a recent study has demonstrated that elevated serum levels at baseline of CD73 correlated with lower response rate, shorter survival and higher rates of progression disease in melanoma patients treated with nivolumab (42).

Besides non-cellular elements which circulate freely in the bloodstream a relevant prognostic role in cancer has certainly been attributed to the deregulation of specific immune subpopulation of cells. This suggests the possibility to evaluate them as predictors of response to immunotherapy as well.

In this regard, Capone and colleagues analyzed peripheral blood mononuclear cells (PBMCs) in samples deriving from 100 nivolumab-treated melanoma patients (43). Among the different subpopulation of immune cells tested, these authors identified CD8+/CD73+ subset of lymphocytes as the strongest associated with worse survival and poor clinical benefits. Indeed, their low baseline percentages were associated with clinical benefits and better survival as compared to non-responsive patients. Even though the biological role of CD8+/CD73+ lymphocytes in the immune response against melanoma cells during nivolumab treatment is still unknown, the expression of CD73 on T cells has been suggested capable of promoting an exhausted phenotype in pre-clinical mouse models of head and neck squamous cell carcinoma (HNSCC) (64). Another class of immune cells associated with the response to immunotherapy in melanoma is myeloid-derived suppressor cells (MDSCs). Ipilimumab-treated melanoma patients with better response to therapy have lower baseline levels of these cells compared to those that do not (non-responders) (44). Importantly, a reduction of MDSCs in the blood also predicts better progression-free survival after neoadjuvant ipilimumab treatment (45).

Finally, the potential consequences of immune-checkpoint inhibitor administration such as the cytotoxic effect as well as local and systemic inflammation should be considered (71). These events are frequently associated with alterations in peripheral blood leukocytes that can be highlighted by evaluating the neutrophil-to-lymphocyte ratio (NLR) (71). Various studies have highlighted that in patients with unresectable stage III-IV melanoma, elevated NLR at baseline was associated with worse overall survival, progression-free survival, and clinical response following ipilimumab and nivolumab treatments (46, 72). Altogether the findings reviewed in this paragraph strongly suggest that liquid biopsies based on the evaluation of cellular and non-cellular elements may be valuable tools in predicting response to immunotherapy in melanoma to the same extent as compared to ctDNA. The results described in this paragraph are schematically summarized in **Table 1**.

## Non Coding RNAs (ncRNAs)

Over the last two decades ncRNAs have emerged as key players in the development of human cancers as well as in the establishment of resistance to several anticancer treatments (73–75). In this review, we will not discuss their biological roles as this topic has already been widely discussed in outstanding recent reviews (76, 77).

It is important to point out tha9t the development of ncRNA signatures in the blood is a more challenging field of research as compared to the other biomarkers already described in the previous sections. Some of the main limitations are due to three issues: 1) their low abundance in body fluids; 2) the difficulty in normalizing the results as there are no suitable endogenous ncRNAs that can be used as “housekeeping” reference analytes at the moment and 3) the great intra-patient variability limiting the possibility of finding consistency between biomarkers identified in different studies (78). However, it has to be considered that thanks to their great stability in body fluids, ncRNAs are emerging in several studies as novel potential biomarkers. A large part of these studies focused on the most abundant class of ncRNAs, namely microRNAs (miRNAs). The stability of these molecules in the blood is preserved by their association with proteins such as AGO1/AGO2, proteolipid complexes and above all extracellular vesicles (EVs) (79, 80).

EVs are broadly classified into four subtypes based upon vesicle size: 1) exosomes (30-150 nm), 2) microvesicles (50-1000 nm), 3) large vesicles (>1000 nm) and 4) apoptotic bodies (>1000 nm) (81). As to metastatic melanoma, most studies have assessed the role of miRNAs as predictors of response to targeted therapies in BRAF-mutant patients (82–85). For example, we have demonstrated that a mini-signature composed of four miRNAs, i.e. miR-199b-5p, miR-204-5p, miR-4443 and miR-4488, is able to distinguish drug-sensitive from drug-resistant patients (83). In contrast, much less is known about miRNAs as biomarkers of response to immunotherapy. For some time, the only data available focused on solid biopsies and provided only indirect information about circulating biomarkers. For example, miR-17-5p levels were shown to be anti-correlated with those of PD-L1 in melanoma tissue biopsies from patients resistant to BRAFi or MEKi therapy (86). Likewise, plasma levels of this miRNA were shown to be higher in patients with PD-L1+ tissues as compared to PD-L1- 9lesions. Along the same line, the expression levels of the oncogenic miR-222 were shown to be higher in solid biopsies from melanoma patients who had no clinical benefit from ipilimumab as compared to patients who responded to such therapy (87). These findings suggest that miR-222 could be a biomarker for predicting response to anti-CTLA-4 checkpoint inhibitors. To the best of our knowledge, the first study demonstrating that a signature of circulating miRNAs is potentially able to predict response to immunotherapy in melanoma was performed by Huber and colleagues (47). These authors showed that a set of 8 miRNAs, i.e. miR-146a, miR-155, miR-125b, miR-100, let-7e, miR-125a, miR-146b, miR-99b are significantly higher in the plasma of patients who did not respond to immunotherapy (i.e. ipilimumab or nivolumab) as compared to responders. Interestingly, the same study also demonstrated that this signature of miRNAs correlated with increased levels of MDSCs in the blood of non-responding patients (47). The subpopulation of immune cells represents a major obstacle to effective immunotherapy in melanoma and is considered a valuable biomarker of response as well (see previous section). Afterwards, miRNA contents of melanoma-derived exosomes were correlated with response to immunotherapy. In taking advantage of flow cytometry and microscopy techniques, it was demonstrated that CD8+ T cells actively internalize specific miRNAs carried by melanoma exosomes (48). Among them, miR-3187-3p, miR-498 and miR-181a/b were found to be able to regulate TCR signaling and TNFa secretion reducing immune response against the tumor. These findings suggest that those miRNAs could be measured in the blood and potentially correlate with the response to immunotherapy in melanoma. Along the same line, a recent comprehensive transcriptomic profiling performed on plasma-derived extracellular vesicles (EVs) from 50 patients with metastatic melanoma underwent immunotherapy and were divided into responders (n= 33) and non-responders (n= 17) (49) 9. Results demonstrated that EVs deriving from non-responding patients are enriched for transcriptional signatures belonging to immune- and tumor-related pathways such as CD1A, MAP2K4, TRBV7-2 and IGFL1. In regards to the miRNA content, authors found some miRNAs enriched in the EVs from non-responding patients, such as miR-551a; in contrast, other miRNAs such as miR-4519 and miR-4674 were enriched in the EVs derived from responding patients (49). Finally, a pilot study identified a signature composed of three circulating miRNAs, namely miR-4649-3p, miR-615-3p and miR-1234-3p as potentially capable of distinguishing melanoma patients who better respond to immunotherapy (ipilimumab, nivolumab, pembrolizumab, or the combination of ipilimumab and nivolumab as first line therapy) (50). In particular, the levels of these miRNAs significantly decreased in the post-treatment samples derived from patients who had a complete response (CR) as compared to patients who progressed from immunotherapy. Interestingly, the increased levels of one of these miRNA, i.e. miR-615-3p showed a superior statistic capability in predicting PD in post-therapy plasma samples as compared to LDH levels (50).

Unlike miRNAs, the other classes of ncRNAs have not been studied as much as circulating biomarkers that predict cancer management and response to therapy. Emerging findings indicate that long non coding RNAs (lncRNAs) and circular RNAs (circRNAs) may be involved in resistance to immunotherapy because they regulate immune cell-specific gene signatures that mediate immune escape (11, 88, 89). However, up until now no studies have investigated their deregulation in liquid biopsies as predictors of response to checkpoint inhibitors in melanoma. So far only one study has tried to correlate lncRNA alterations to the prediction of immunotherapy response. This study used 9data available from The Cancer Genome Atlas (TCGA) belonging to bladder cancer and melanoma patients treated with immunotherapy for identifying lncRNA profiles associated with immune response (89). These bioinformatics analyses led to identifying a signature composed of 49 lncRNAs that are potentially capable of distinguishing cancer patients who benefit from immunotherapy. Among them, the low expression levels of a specific lncRNA, called NKILA were found to be able to distinguish patients who respond to immunotherapy as compared to those that do not (non-responders). These initial findings warrant future validation in additional confirmatory studies.

Altogether the results of this section highlight the potential role of ncRNAs as predictors of response to immunotherapy in melanoma. However, it is evident that compared to CTCs and ctDNA for example, their development as robust biomarkers is still in its infant stage.

The main findings described in the above paragraphs are schematically summarized in **Table 1**.

## Final Considerations

One major therapeutic breakthrough over the past ten years in medical oncology has been the introduction of immunotherapy with checkpoint inhibitors targeting CTLA-4 and PD-1/PD-L1 axis (7). After its initial suc9cess in the treatment of metastatic melanoma, the clinical application of checkpoint inhibitors has rapidly spread to the majority of cancer cases with varying degrees of success. Among several cancer types, melanoma remains one of the most positively impacted by the use of these molecules, most likely because of its high mutational rate as well as to the frequent generation of an inflammatory microenvironment which together help establish appropriate conditions for the immune-system to respond (90, 91). In stage IV melanoma, five year overall survival rates obtained with the combination of ipilimumab and nivolumab are currently up to 52% (92). Three Phase III combination studies in the subset of patients with BRAF V600 mutation using a BRAF plus a MEK inhibitor and immunotherapy with anti-PD-1 or anti-PD-L1 have been carried out (93–95). Although we have not yet obtained data about long term outcomes from these studies, in two out of three trials, progression free survival did not improve in the combo therapy arm of immunotherapy plus targeted therapy over targeted therapy alone and in the third trial there was a modest improvement. Hence, the issue still remains that a significant proportion of patients do not benefit from existing therapies both alone or in combination.

**Figure 2:**
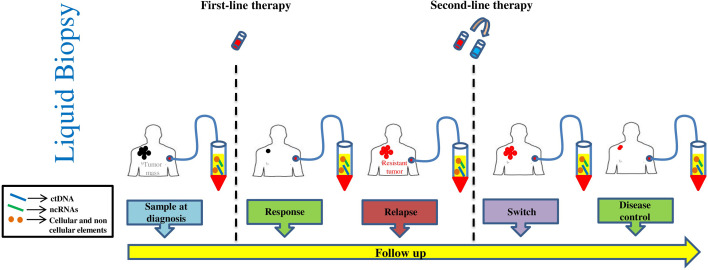
Schematic representation of liquid biopsy sampling.

Significant efforts are being devoted to understanding the biological and immunological basis of drug resistance and several clinical trials are being conducted with combinations of additional checkpoint inhibitors i9n the attempt to improve response rates (94, 95). At the same time, it is important to develop biomarkers capable of identifying responders from non-responders to current therapy. The main reasons being the elevated costs and the high rate of serious immunological adverse events, in particular those observed with the combination of anti-CTLA4 and anti-PD-1/anti-PD-L1 antibodies (10). In this regard, a significant contribution has been provided by the discovery that the combination of interferon signature and mutational burden in tumor biopsies is a better, albeit not absolute predictor, of response than the simple detection of PD-L1 (96). In principle however, an ideal predictive biomarker should have the following features: a) require minimally invasive procedures to be measured, b) allow real-time longitudinal monitoring during the course of therapy and c) be easy to be measured and standardized. LBs using blood samples provide the ideal solution (**Figure 2**). This approach has the potential to overcome the shortcomings of repeated re-biopsies of tumors that are often difficult to obtain (97). Furthermore, the issue with sensitivity of liquid biopsies has been solved thanks to major technological advancements reached in the last few years. As it has been reviewed in this paper, several analytes can be detected in the blood: 1) nucleic acids like ctDNA and ncRNAs, 2) cellular elements like CTCs and immune cells and 3) soluble factors like cytokines and enzymes. Clinical data are more mature with ctDNA based assays and is relevant to consider that a ctDNA assay based on the simultaneous measure of 16 different patient-specific tumor mutations in the blood, has recently been approved in the US by Medicare to monitor minimal residual disease in Stage II and III colorectal cancer and to guide therapeutic decisions (Signatera™ MRD Test) (98, 99). Very promising data have been obtained using the same technology for predicting response to pembrolizumab in patients with various solid cancers (31). Hence, we may expect in future years a prominent increase in ctDNA validation studies and registratio9n of several blood based assays. The outcome of these studies will allow to set up new companion diagnostics able to direct/guide precision medicine in oncology.

## Author Contributions

LF conceptualized and revised the manuscript. CFR did figures and drafted the manuscript. DL did table and drafted the manuscript. VC did figures and revised bibliography. AC helped to draft the manuscript. GC supervised and revised the manuscript and provided critical suggestions. RM revised the manuscript and provided critical suggestions. All authors have read and agreed to the published version of the manuscript. All authors contributed to the article and approved the submitted version.

## Funding

This work was supported by: 1) Italian Association for Cancer Research (AIRC) grants IG15216 to GC and IG17009 to RM; 2) the Lazioinova grant 2018 n.85-2017-13750 to RM; 3) PRIN Bando 2017 (Prot. 2017HWTP2K) to GC and RM.

## Conflict of Interest

The authors declare that the research was conducted in the absence of any commercial or financial relationships that could be construed as a potential conflict of interest.

## Abbreviations

LB, liquid biopsy; PD-1, Programmed Cell Death Protein 1; PD-L1, Programmed Cell Death Ligand 1; ctDNA, circulating tumor DNA; ctRNA, circulating tumor RNA; CTCs, circulating tumor cell; EV, and extracellular vesicle; ICB, immune checkpoint blockers; cfDNA, cell-free DNA; ddPCR, Droplet Digital PCR; TMB, tumor mutation burden; OS, overall survival; PFS, progression free survival; MST, mixed solid tumors; LDH, Lactate Dehydrogenase; NLR, neutrophil-to-lymphocyte ratio; PBMC, peripheral blood mononuclear cell; HNSCC, head and neck squamous cell carcinoma; MDSC, myeloid-derived suppressor cell.

## References

[1] Rodríguez-Cerdeira C Carnero Gregorio M López-Barcenas A Sánchez-Blanco E Sánchez-Blanco B Fabbrocini G. Advances in Immunotherapy for Melanoma: A Comprehensive Review. Mediators Inflamm (2017) 2017:3264217. 10.1155/2017/3264217 28848246 PMC5564072

[2] Jamal-Hanjani M Quezada SA Larkin J Swanton C. Translational implications of tumor heterogeneity. Clin Cancer Res (2015) 21:1258–66. 10.1158/1078-0432.CCR-14-1429 PMC4374162 25770293

[3] O’Donnell JS Teng MWL Smyth MJ. Cancer immunoediting and resistance to T cell-based immunotherapy. Nat Rev Clin Oncol (2019) 16:151–67. 10.1038/s41571-018-0142-8 30523282

[4] Bruschini S Ciliberto G Mancini R. The emerging role of cancer cell plasticity and cell-cycle quiescence in immune escape. Cell Death Dis (2020) 11:471. 10.1038/s41419-020-2669-8 32555172 PMC7303167

[5] Bai X Fisher DE Flaherty KT. Cell-state dynamics and therapeutic resistance in melanoma from the perspective of MITF and IFNγ pathways. Nat Rev Clin Oncol (2019) 16:549–62. 10.1038/s41571-019-0204-6 PMC7185899 30967646

[6] Tucci M Passarelli A Mannavola F Felici C Stucci LS Cives M. Immune System Evasion as Hallmark of Melanoma Progression: The Role of Dendritic Cells. Front Oncol (2019) 9:1148. 10.3389/fonc.2019.01148 31750245 PMC6848379

[7] Luke JJ Flaherty KT Ribas A Long GV. Targeted agents and immunotherapies: optimizing outcomes in melanoma. Nat Rev Clin Oncol (2017) 14:463–82. 10.1038/nrclinonc.2017.43 28374786

[8] Simeone E Grimaldi AM Festino L Trojaniello C Vitale MG Vanella V. Immunotherapy in metastatic melanoma: a novel scenario of new toxicities and their management. Melanoma Manage (2019) 6:MMT30. 10.2217/mmt-2019-0005 PMC6920742 31871619

[9] Topalian SL Taube JM Anders RA Pardoll DM. Mechanism-driven biomarkers to guide immune checkpoint blockade in cancer therapy. Nat Rev Cancer (2016) 16:275–87. 10.1038/nrc.2016.36 PMC5381938 27079802

[10] Larkin J Hodi FS Wolchok JD. Combined Nivolumab and Ipilimumab or Monotherapy in Untreated Melanoma. N Engl J Med (2015) 373:1270–1. 10.1056/NEJMc1509660 26398076

[11] Fattore L Mancini R Ascierto PA Ciliberto G. The potential of BRAF-associated non-coding RNA as a therapeutic target in melanoma. Expert Opin Ther Targets (2019) 23:53–68. 10.1080/14728222.2019.1554057 30507327

[12] Imbert C Montfort A Fraisse M Marcheteau E Gilhodes J Martin E. Resistance of melanoma to immune checkpoint inhibitors is overcome by targeting the sphingosine kinase-1. Nat Commun (2020) 11:437. 10.1038/s41467-019-14218-7 31974367 PMC6978345

[13] Sharma P Hu-Lieskovan S Wargo JA Ribas A. Primary, Adaptive, and Acquired Resistance to Cancer Immunotherapy. Cell (2017) 168:707–23. 10.1016/j.cell.2017.01.017 PMC5391692 28187290

[14] Sun J Carr MJ Khushalani NI. Principles of Targeted Therapy for Melanoma. Surg Clin North Am (2020) 100:175–88. 10.1016/j.suc.2019.09.013 31753111

[15] Huynh K Hoon DSB. Liquid Biopsies for Assessing Metastatic Melanoma Progression. Crit Rev Oncog (2016) 21:141–54. 10.1615/CritRevOncog.2016016075 PMC5326707 27481010

[16] De Rubis G Rajeev Krishnan S Bebawy M. Liquid Biopsies in Cancer Diagnosis, Monitoring, and Prognosis. Trends Pharmacol Sci (2019) 40:172–86. 10.1016/j.tips.2019.01.006 30736982

[17] Corcoran RB. Liquid biopsy versus tumor biopsy for clinical-trial recruitment. Nat Med (2020) 26:1815–6. 10.1038/s41591-020-01169-6 33230339

[18] Qi Z-H Xu H-X Zhang S-R Xu J-Z Li S Gao H-L. The Significance of Liquid Biopsy in Pancreatic Cancer. J Cancer (2018) 9:3417–26. 10.7150/jca.24591 PMC6160675 30271504

[19] Lim SY Lee JH Diefenbach RJ Kefford RF Rizos H. Liquid biomarkers in melanoma: detection and discovery. Mol Cancer (2018) 17:8. 10.1186/s12943-018-0757-5 29343260 PMC5772714

[20] Heitzer E. Circulating Tumor DNA for Modern Cancer Management. Clin Chem (2019) 66:143–5. 10.1373/clinchem.2019.304774 31672857

[21] Papadopoulos N. Pathophysiology of ctDNA Release into the Circulation and Its Characteristics: What Is Important for Clinical Applications. Recent Results Cancer Res (2020) 215:163–80. 10.1007/978-3-030-26439-0_9 31605229

[22] Song Y Hu C Xie Z Wu L Zhu Z Rao C. Circulating tumor DNA clearance predicts prognosis across treatment regimen in a large real-world longitudinally monitored advanced non-small cell lung cancer cohort. Transl Lung Cancer Res (2020) 9:269–79. 10.21037/tlcr.2020.03.17 PMC7225135 32420066

[23] Calapre L Warburton L Millward M Gray ES. Circulating tumour DNA (ctDNA) as a biomarker in metachronous melanoma and colorectal cancer- a case report. BMC Cancer (2019) 19:1109. 10.1186/s12885-019-6336-3 31727009 PMC6857141

[24] Gray JE Okamoto I Sriuranpong V Vansteenkiste J Imamura F Lee JS. Tissue and Plasma EGFR Mutation Analysis in the FLAURA Trial: Osimertinib versus Comparator EGFR Tyrosine Kinase Inhibitor as First-Line Treatment in Patients with EGFR-Mutated Advanced Non-Small Cell Lung Cancer. Clin Cancer Res (2019) 25:6644–52. 10.1158/1078-0432.CCR-19-1126 PMC7209579 31439584

[25] Barbany G Arthur C Liedén A Nordenskjöld M Rosenquist R Tesi B. Cell-free tumour DNA testing for early detection of cancer - a potential future tool. J Intern Med (2019) 286:118–36. 10.1111/joim.12897 30861222

[26] Paik PK Felip E Veillon R Sakai H Cortot AB Garassino MC. Tepotinib in Non-Small-Cell Lung Cancer with MET Exon 14 Skipping Mutations. N Engl J Med (2020) 383:931–43. 10.1056/NEJMoa2004407 PMC8422679 32469185

[27] Forschner A Battke F Hadaschik D Schulze M Weißgraeber S Han C-T. Tumor mutation burden and circulating tumor DNA in combined CTLA-4 and PD-1 antibody therapy in metastatic melanoma - results of a prospective biomarker study. J Immunother cancer. (2019) 7:180. 10.1186/s40425-019-0659-0 31300034 PMC6625062

[28] Goodman AM Kato S Bazhenova L Patel SP Frampton GM Miller V. Tumor Mutational Burden as an Independent Predictor of Response to Immunotherapy in Diverse Cancers. Mol Cancer Ther (2017) 16:2598–608. 10.1158/1535-7163.MCT-17-0386 PMC5670009 28835386

[29] Seremet T Jansen Y Planken S Njimi H Delaunoy M El Housni H. Undetectable circulating tumor DNA (ctDNA) levels correlate with favorable outcome in metastatic melanoma patients treated with anti-PD1 therapy. J Transl Med (2019) 17:303. 10.1186/s12967-019-2051-8 31488153 PMC6727487

[30] Lee JH Long GV Boyd S Lo S Menzies AM Tembe V. Circulating tumour DNA predicts response to anti-PD1 antibodies in metastatic melanoma. Ann Oncol (2017) 28:1130–6. 10.1093/annonc/mdx026 28327969

[31] Bratman SV Yang SYC Iafolla MAJ Liu Z Hansen AR Bedard PL. Personalized circulating tumor DNA analysis as a predictive biomarker in solid tumor patients treated with pembrolizumab. Nat Cancer (2020) 1:873–81. 10.1038/s43018-020-0096-5 35121950

[32] Ashida A Sakaizawa K Uhara H Okuyama R. Circulating Tumour DNA for Monitoring Treatment Response to Anti-PD-1 Immunotherapy in Melanoma Patients. Acta Derm Venereol (2017) 97:1212–8. 10.2340/00015555-2748 28681063

[33] Gray ES Rizos H Reid AL Boyd SC Pereira MR Lo J. Circulating tumor DNA to monitor treatment response and detect acquired resistance in patients with metastatic melanoma. Oncotarget (2015) 6:42008–18. 10.18632/oncotarget.5788 PMC4747205 26524482

[34] Schreuer M Meersseman G Van Den Herrewegen S Jansen Y Chevolet I Bott A. Quantitative assessment of BRAF V600 mutant circulating cell-free tumor DNA as a tool for therapeutic monitoring in metastatic melanoma patients treated with BRAF/MEK inhibitors. J Transl Med (2016) 14:95. 10.1186/s12967-016-0852-6 27095081 PMC4837559

[35] Hong X Sullivan RJ Kalinich M Kwan TT Giobbie-Hurder A Pan S. Molecular signatures of circulating melanoma cells for monitoring early response to immune checkpoint therapy. Proc Natl Acad Sci U S A (2018) 115:2467–72. 10.1073/pnas.1719264115 PMC5877960 29453278

[36] Lin SY Chang S-C Lam S Irene Ramos R Tran K Ohe S. Prospective Molecular Profiling of Circulating Tumor Cells from Patients with Melanoma Receiving Combinatorial Immunotherapy. Clin Chem (2020) 66:169–77. 10.1373/clinchem.2019.307140 PMC7193771 31672856

[37] Khattak MA Reid A Freeman J Pereira M McEvoy A Lo J. PD-L1 Expression on Circulating Tumor Cells May Be Predictive of Response to Pembrolizumab in Advanced Melanoma: Results from a Pilot Study. Oncologist (2019) 25:e520–7. 10.1634/theoncologist.2019-0557 PMC7066715 32162809

[38] Zhou J Mahoney KM Giobbie-Hurder A Zhao F Lee S Liao X. Soluble PD-L1 as a Biomarker in Malignant Melanoma Treated with Checkpoint Blockade. Cancer Immunol Res (2017) 5:480–92. 10.1158/2326-6066.CIR-16-0329 PMC5642913 28522460

[39] Weber JS Sznol M Sullivan RJ Blackmon S Boland G Kluger HM. A Serum Protein Signature Associated with Outcome after Anti-PD-1 Therapy in Metastatic Melanoma. Cancer Immunol Res (2018) 6:79–86. 10.1158/2326-6066.CIR-17-0412 29208646

[40] Lim SY Lee JH Gide TN Menzies AM Guminski A Carlino MS. Circulating Cytokines Predict Immune-Related Toxicity in Melanoma Patients Receiving Anti-PD-1-Based Immunotherapy. Clin Cancer Res (2019) 25:1557–63. 10.1158/1078-0432.CCR-18-2795 30409824

[41] Sanmamed MF Perez-Gracia JL Schalper KA Fusco JP Gonzalez A Rodriguez-Ruiz ME. Changes in serum interleukin-8 (IL-8) levels reflect and predict response to anti-PD-1 treatment in melanoma and non-small-cell lung cancer patients. Ann Oncol (2017) 28:1988–95. 10.1093/annonc/mdx190 PMC5834104 28595336

[42] Morello S Capone M Sorrentino C Giannarelli D Madonna G Mallardo D. Soluble CD73 as biomarker in patients with metastatic melanoma patients treated with nivolumab. J Transl Med (2017) 15:244. 10.1186/s12967-017-1348-8 29202855 PMC5716054

[43] Capone M Fratangelo F Giannarelli D Sorrentino C Turiello R Zanotta S. Frequency of circulating CD8+CD73+T cells is associated with survival in nivolumab-treated melanoma patients. J Transl Med (2020) 18:121. 10.1186/s12967-020-02285-0 32160899 PMC7065327

[44] Meyer C Cagnon L Costa-Nunes CM Baumgaertner P Montandon N Leyvraz L. Frequencies of circulating MDSC correlate with clinical outcome of melanoma patients treated with ipilimumab. Cancer Immunol Immunother (2014) 63:247–57. 10.1007/s00262-013-1508-5 PMC11029062 24357148

[45] Tarhini AA Edington H Butterfield LH Lin Y Shuai Y Tawbi H. Immune monitoring of the circulation and the tumor microenvironment in patients with regionally advanced melanoma receiving neoadjuvant ipilimumab. PLoS One (2014) 9:e87705. 10.1371/journal.pone.0087705 24498358 PMC3912016

[46] Capone M Giannarelli D Mallardo D Madonna G Festino L Grimaldi AM. Baseline neutrophil-to-lymphocyte ratio (NLR) and derived NLR could predict overall survival in patients with advanced melanoma treated with nivolumab. J Immunother Cancer (2018) 6:74. 10.1186/s40425-018-0383-1 30012216 PMC6048712

[47] Huber V Vallacchi V Fleming V Hu X Cova A Dugo M. Tumor-derived microRNAs induce myeloid suppressor cells and predict immunotherapy resistance in melanoma. J Clin Invest (2018) 128:5505–16. 10.1172/JCI98060 PMC6264733 30260323

[48] Vignard V Labbé M Marec N André-Grégoire G Jouand N Fonteneau J-F. MicroRNAs in Tumor Exosomes Drive Immune Escape in Melanoma. Cancer Immunol Res (2020) 8:255–67. 10.1158/2326-6066.CIR-19-0522 31857348

[49] Shi A Kasumova GG Michaud WA Cintolo-Gonzalez J Diaz-Martinez M Ohmura J. Plasma-derived extracellular vesicle analysis and deconvolution enable prediction and tracking of melanoma checkpoint blockade outcome. Sci Adv (2020) 6. 10.1126/sciadv.abb3461 PMC7673759 33188016

[50] Bustos MA Gross R Rahimzadeh N Cole H Tran LT Tran KD. A Pilot Study Comparing the Efficacy of Lactate Dehydrogenase Levels Versus Circulating Cell-Free microRNAs in Monitoring Responses to Checkpoint Inhibitor Immunotherapy in Metastatic Melanoma Patients. Cancers (Basel) (2020) 12. 10.3390/cancers12113361 PMC7696545 33202891

[51] Fabisiewicz A Grzybowska E. CTC clusters in cancer progression and metastasis. Med Oncol (2017) 34:12. 10.1007/s12032-016-0875-0 28012133

[52] Attard G de Bono JS. Utilizing circulating tumor cells: challenges and pitfalls. Curr Opin Genet Dev (2011) 21:50–8. 10.1016/j.gde.2010.10.010 21112767

[53] Gkountela S Castro-Giner F Szczerba BM Vetter M Landin J Scherrer R. Circulating Tumor Cell Clustering Shapes DNA Methylation to Enable Metastasis Seeding. Cell (2019) 176:98–112.e14. 10.1016/j.cell.2018.11.046 30633912 PMC6363966

[54] Ruggiero CF Malpicci D Fattore L Madonna G Vanella V Mallardo D. ErbB3 Phosphorylation as Central Event in Adaptive Resistance to Targeted Therapy in Metastatic Melanoma: Early Detection in CTCs during Therapy and Insights into Regulation by Autocrine Neuregulin. Cancers (Basel) (2019) 11. 10.3390/cancers11101425 PMC6826737 31557826

[55] Marsavela G Aya-Bonilla CA Warkiani ME Gray ES Ziman M. Melanoma circulating tumor cells: Benefits and challenges required for clinical application. Cancer Lett (2018) 424:1–8. 10.1016/j.canlet.2018.03.013 29548820

[56] Bankó P Lee SY Nagygyörgy V Zrínyi M Chae CH Cho DH. Technologies for circulating tumor cell separation from whole blood. J Hematol Oncol (2019) 12:48. 10.1186/s13045-019-0735-4 31088479 PMC6518774

[57] Schadendorf D Long GV Stroiakovski D Karaszewska B Hauschild A Levchenko E. Three-year pooled analysis of factors associated with clinical outcomes across dabrafenib and trametinib combination therapy phase 3 randomised trials. Eur J Cancer (2017) 82:45–55. 10.1016/j.ejca.2017.05.033 28648698

[58] Hodi FS Chiarion-Sileni V Gonzalez R Grob J-J Rutkowski P Cowey CL. Nivolumab plus ipilimumab or nivolumab alone versus ipilimumab alone in advanced melanoma (CheckMate 067): 4-year outcomes of a multicentre, randomised, phase 3 trial. Lancet Oncol (2018) 19:1480–92. 10.1016/S1470-2045(18)30700-9 30361170

[59] Gandini S Massi D Mandalà M. PD-L1 expression in cancer patients receiving anti PD-1/PD-L1 antibodies: A systematic review and meta-analysis. Crit Rev Oncol Hematol (2016) 100:88–98. 10.1016/j.critrevonc.2016.02.001 26895815

[60] Nowicki TS Hu-Lieskovan S Ribas A. Mechanisms of Resistance to PD-1 and PD-L1 Blockade. Cancer J (2018) 24:47–53. 10.1097/PPO.0000000000000303 29360728 PMC5785093

[61] Guibert N Delaunay M Lusque A Boubekeur N Rouquette I Clermont E. PD-L1 expression in circulating tumor cells of advanced non-small cell lung cancer patients treated with nivolumab. Lung Cancer (2018) 120:108–12. 10.1016/j.lungcan.2018.04.001 29748004

[62] Roscilli G De Vitis C Ferrara FF Noto A Cherubini E Ricci A. Human lung adenocarcinoma cell cultures derived from malignant pleural effusions as model system to predict patients chemosensitivity. J Transl Med (2016) 14:61. 10.1186/s12967-016-0816-x 26928703 PMC4772534

[63] Gu D Ao X Yang Y Chen Z Xu X. Soluble immune checkpoints in cancer: production, function and biological significance. J Immunother Cancer (2018) 6:132. 10.1186/s40425-018-0449-0 30482248 PMC6260693

[64] Frigola X Inman BA Lohse CM Krco CJ Cheville JC Thompson RH. Identification of a soluble form of B7-H1 that retains immunosuppressive activity and is associated with aggressive renal cell carcinoma. Clin Cancer Res (2011) 17:1915–23. 10.1158/1078-0432.CCR-10-0250 PMC3241002 21355078

[65] Rossille D Gressier M Damotte D Maucort-Boulch D Pangault C Semana G. High level of soluble programmed cell death ligand 1 in blood impacts overall survival in aggressive diffuse large B-Cell lymphoma: results from a French multicenter clinical trial. Leukemia (2014) 28:2367–75. 10.1038/leu.2014.137 24732592

[66] Wang L Wang H Chen H Wang W Chen X-Q Geng Q-R. Serum levels of soluble programmed death ligand 1 predict treatment response and progression free survival in multiple myeloma. Oncotarget (2015) 6:41228–36. 10.18632/oncotarget.5682 PMC4747402 26515600

[67] Lu D Ni Z Liu X Feng S Dong X Shi X. Beyond T Cells: Understanding the Role of PD-1/PD-L1 in Tumor-Associated Macrophages. J Immunol Res (2019) 2019:1919082. 10.1155/2019/1919082 31781673 PMC6875348

[68] Allard B Longhi MS Robson SC Stagg J. The ectonucleotidases CD39 and CD73: Novel checkpoint inhibitor targets. Immunol Rev (2017) 276:121–44. 10.1111/imr.12528 PMC5338647 28258700

[69] Neo SY Yang Y Record J Ma R Chen X Chen Z. CD73 immune checkpoint defines regulatory NK cells within the tumor microenvironment. J Clin Invest (2020) 130:1185–98. 10.1172/JCI128895 PMC7269592 31770109

[70] Wang R Zhang Y Lin X Gao Y Zhu Y. Prognositic value of CD73-adenosinergic pathway in solid tumor: A meta-analysis and systematic review. Oncotarget (2017) 8:57327–36. 10.18632/oncotarget.16905 PMC5593644 28915673

[71] Zahorec R. Ratio of neutrophil to lymphocyte counts–rapid and simple parameter of systemic inflammation and stress in critically ill. Bratisl Lek Listy (2001) 102:5–14. 11723675

[72] Weide B Martens A Hassel JC Berking C Postow MA Bisschop K. Baseline Biomarkers for Outcome of Melanoma Patients Treated with Pembrolizumab. Clin Cancer Res (2016) 22:5487–96. 10.1158/1078-0432.CCR-16-0127 PMC5572569 27185375

[73] Fattore L Costantini S Malpicci D Ruggiero CF Ascierto PA Croce CM. MicroRNAs in melanoma development and resistance to target therapy. Oncotarget (2017) 8:22262–78. 10.18632/oncotarget.14763 PMC5400662 28118616

[74] Fattore L Sacconi A Mancini R Ciliberto G. MicroRNA-driven deregulation of cytokine expression helps development of drug resistance in metastatic melanoma. Cytokine Growth Factor Rev (2017) 36:39–48. 10.1016/j.cytogfr.2017.05.003 28551321

[75] Varrone F Caputo E. The miRNAs Role in Melanoma and in Its Resistance to Therapy. Int J Mol Sci (2020) 21. 10.3390/ijms21030878 PMC7037367 32013263

[76] Panni S Lovering RC Porras P Orchard S. Non-coding RNA regulatory networks. Biochim Biophys Acta Gene Regul Mech (2020) 1863:194417. 10.1016/j.bbagrm.2019.194417 31493559

[77] Acunzo M Romano G Wernicke D Croce CM. MicroRNA and cancer–a brief overview. Adv Biol Regul (2015) 57:1–9. 10.1016/j.jbior.2014.09.013 25294678

[78] Mumford SL Towler BP Pashler AL Gilleard O Martin Y Newbury SF. Circulating MicroRNA Biomarkers in Melanoma: Tools and Challenges in Personalised Medicine. Biomolecules (2018) 8. 10.3390/biom8020021 PMC6022922 29701682

[79] Salehi M Sharifi M. Exosomal miRNAs as novel cancer biomarkers: Challenges and opportunities. J Cell Physiol (2018) 233:6370–80. 10.1002/jcp.26481 PMC13482446 29323722

[80] Cui M Wang H Yao X Zhang D Xie Y Cui R. Circulating MicroRNAs in Cancer: Potential and Challenge. Front Genet (2019) 10:626. 10.3389/fgene.2019.00626 31379918 PMC6656856

[81] Butler JT Abdelhamed S Kurre P. Extracellular vesicles in the hematopoietic microenvironment. Haematologica (2018) 103:382–94. 10.3324/haematol.2017.183335 PMC5830368 29439185

[82] Fattore L Mancini R Acunzo M Romano G Laganà A Pisanu ME. miR-579-3p controls melanoma progression and resistance to target therapy. Proc Natl Acad Sci U S A (2016) 113:E5005–13. 10.1073/pnas.1607753113 PMC5003278 27503895

[83] Fattore L Ruggiero CF Pisanu ME Liguoro D Cerri A Costantini S. Reprogramming miRNAs global expression orchestrates development of drug resistance in BRAF mutated melanoma. Cell Death Differ (2019) 26:1267–82. 10.1038/s41418-018-0205-5 PMC6748102 30254376

[84] Caporali S Amaro A Levati L Alvino E Lacal PM Mastroeni S. miR-126-3p down-regulation contributes to dabrafenib acquired resistance in melanoma by up-regulating ADAM9 and VEGF-A. J Exp Clin Cancer Res (2019) 38:272. 10.1186/s13046-019-1238-4 31227006 PMC6588909

[85] Tupone MG D’Aguanno S Di Martile M Valentini E Desideri M Trisciuoglio D. microRNA-378a-5p iS a novel positive regulator of melanoma progression. Oncogenesis (2020) 9:22. 10.1038/s41389-020-0203-6 32060259 PMC7021836

[86] Audrito V Serra S Stingi A Orso F Gaudino F Bologna C. PD-L1 up-regulation in melanoma increases disease aggressiveness and is mediated through miR-17-5p. Oncotarget (2017) 8:15894–911. 10.18632/oncotarget.15213 PMC5362532 28199980

[87] Galore-Haskel G Nemlich Y Greenberg E Ashkenazi S Hakim M Itzhaki O. A novel immune resistance mechanism of melanoma cells controlled by the ADAR1 enzyme. Oncotarget (2015) 6:28999–9015. 10.18632/oncotarget.4905 PMC4745707 26338962

[88] Xu Z Li P Fan L Wu M. The Potential Role of circRNA in Tumor Immunity Regulation and Immunotherapy. Front Immunol (2018) 9:9. 10.3389/fimmu.2018.00009 29403493 PMC5786515

[89] Yu Y Zhang W Li A Chen Y Ou Q He Z. Association of Long Noncoding RNA Biomarkers With Clinical Immune Subtype and Prediction of Immunotherapy Response in Patients With Cancer. JAMA Netw Open (2020) 3:e202149–e202149. 10.1001/jamanetworkopen.2020.2149 32259264 PMC7139278

[90] Grzywa TM Paskal W Włodarski PK. Intratumor and Intertumor Heterogeneity in Melanoma. Transl Oncol (2017) 10:956–75. 10.1016/j.tranon.2017.09.007 PMC5671412 29078205

[91] Di Martile M Farini V Consonni FM Trisciuoglio D Desideri M Valentini E. Melanoma-specific bcl-2 promotes a protumoral M2-like phenotype by tumor-associated macrophages. J Immunother Cancer (2020) 8. 10.1136/jitc-2019-000489 PMC7254128 32269145

[92] Larkin J Chiarion-Sileni V Gonzalez R Grob J-J Rutkowski P Lao CD. Five-Year Survival with Combined Nivolumab and Ipilimumab in Advanced Melanoma. N Engl J Med (2019) 381:1535–46. 10.1056/NEJMoa1910836 31562797

[93] Ascierto PA Ferrucci PF Stephens R Del Vecchio M Atkinson V Schmidt H. KEYNOTE-022 Part 3: phase II randomized study of 1L dabrafenib (D) and trametinib (T) plus pembrolizumab (Pembro) or placebo (PBO) for BRAF-mutant advanced melanoma. Ann Oncol (2018) 29:viii442–. 10.1093/annonc/mdy289

[94] Gutzmer R Stroyakovskiy D Gogas H Robert C Lewis K Protsenko S. Atezolizumab, vemurafenib, and cobimetinib as first-line treatment for unresectable advanced BRAF^V600^ mutation-positive melanoma (IMspire150): primary analysis of the randomised, double-blind, placebo-controlled, phase 3 trial. Lancet (2020) 395:1835–44. 10.1016/S0140-6736(20)30934-X 32534646

[95] Nathan P Dummer R Long GV Ascierto PA Tawbi HA Robert C. LBA43 Spartalizumab plus dabrafenib and trametinib (Sparta-DabTram) in patients (pts) with previously untreated BRAF V600–mutant unresectable or metastatic melanoma: Results from the randomized part 3 of the phase III COMBI-i trial. Ann Oncol (2020) 31:S1172. 10.1016/j.annonc.2020.08.2273

[96] Cristescu R Mogg R Ayers M Albright A Murphy E Yearley J. Pan-tumor genomic biomarkers for PD-1 checkpoint blockade{\textendash}based immunotherapy. Science (2018) 362. 10.1126/science.aar3593 PMC6718162 30309915

[97] Fattore L Ruggiero CF Liguoro D Mancini R Ciliberto G. Single cell analysis to dissect molecular heterogeneity and disease evolution in metastatic melanoma. Cell Death Dis (2019) 10. 10.1038/s41419-019-2048-5 PMC6823362 31672982

[98] Reinert T Henriksen TV Christensen E Sharma S Salari R Sethi H. Analysis of Plasma Cell-Free DNA by Ultradeep Sequencing in Patients With Stages I to III Colorectal Cancer. JAMA Oncol (2019) 5:1124–31. 10.1001/jamaoncol.2019.0528 PMC6512280 31070691

[99] Christensen E Birkenkamp-Demtröder K Sethi H Shchegrova S Salari R Nordentoft I. Early Detection of Metastatic Relapse and Monitoring of Therapeutic Efficacy by Ultra-Deep Sequencing of Plasma Cell-Free DNA in Patients With Urothelial Bladder Carcinoma. J Clin Oncol (2019) 37:1547–57. 10.1200/JCO.18.02052 31059311

